# A challenging case of fungating malignant melanoma

**DOI:** 10.11604/pamj.2018.31.204.16050

**Published:** 2018-11-22

**Authors:** Krishna Mohan Baradhi

**Affiliations:** 1 Division of Nephrology, University of Oklahoma, Tulsa, USA

**Keywords:** Melanoma, UV light, immunotherapy, skin cancer

## Image in medicine

We present the case of a 43-yearr-old male present with left arm pain from a football-sized mass over his left shoulder and arm. He had a history of 3cm melanoma on his left back 3 years ago and underwent resection and adjuvant radiation. However, he had recurrence of melanoma a year later with metastases to the left supra clavicular lymph nodes. He underwent further resection followed by multiple regimens of chemotherapy, which included vemurafenib, dabrafenib and trametinib. Unfortunately, his melanoma was aggressive and remained refractory to the currently available standard therapies. Physical examination as visualized in the figure. The patient eventually opted for a palliative total upper extremity amputation including shoulder joint. World Health Organization estimates around 132,000 new cases of melanoma are diagnosed globally every year. Melanoma is the fifth leading cancer in males and seventh leading cancer in females in the United States. Melanoma is deadliest of all skin cancers and its incidence continues to rise. While recent targeted immunotherapies have improved the survival period of melanoma, metastatic melanoma can be fatal even with the currently available therapies. The single most important preventive measure is to avoid excessive ultraviolet radiation and there should be heightened awareness of its risk factors among both clinicians and patients. Patient's education on prevention and early recognition are paramount for battling melanoma and for better outcomes. Novel biological therapies have undoubtedly made substantial progress and will likely change the landscape in the treatment of melanoma in the near future.

**Figure 1:**
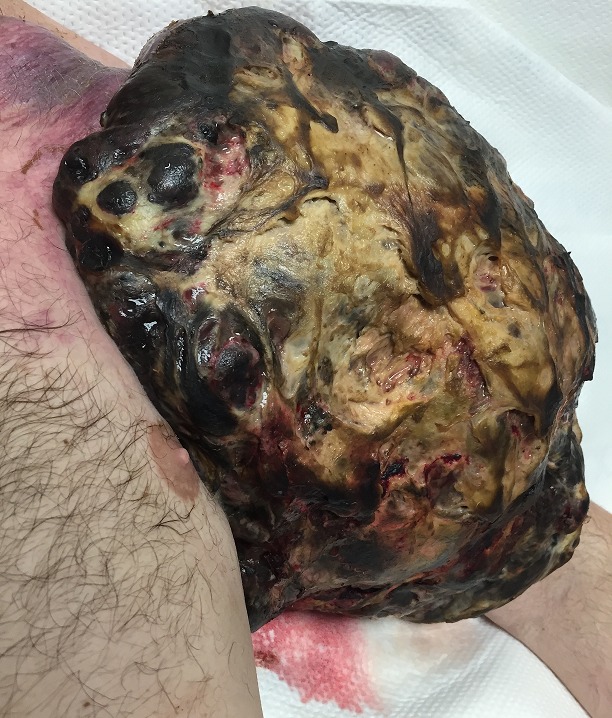
Malignant melanoma 18 x 10 cm along the lateral aspect of left nipple extending cranio-caudally from inferior aspect of shoulder to elbow joint

